# Independent centromere formation in a capricious, gene-free domain of chromosome 13q21 in Old World monkeys and pigs

**DOI:** 10.1186/gb-2006-7-10-r91

**Published:** 2006-10-13

**Authors:** Maria Francesca Cardone, Alicia Alonso, Michele Pazienza, Mario Ventura, Gabriella Montemurro, Lucia Carbone, Pieter J de Jong, Roscoe Stanyon, Pietro D'Addabbo, Nicoletta Archidiacono, Xinwei She, Evan E Eichler, Peter E Warburton, Mariano Rocchi

**Affiliations:** 1 Department of Genetics and Microbiology, University of Bari, Bari, Italy; 2 Department of Human Genetics, Mount Sinai School of Medicine, New York, New York 10029, USA; 3 Children's Hospital Oakland Research Institute, Oakland, California 94609, USA; 4 Department of Animal Biology and Genetics 'Leo Pardi', University of Florence, Florence, Italy; 5 Howard Hughes Medical Institute, Department of Genome Sciences, University of Washington School of Medicine, Seattle, Washington 98195, USA

## Abstract

**Background:**

Evolutionary centromere repositioning and human analphoid neocentromeres occurring in clinical cases are, very likely, two stages of the same phenomenon whose properties still remain substantially obscure. Chromosome 13 is the chromosome with the highest number of neocentromeres. We reconstructed the mammalian evolutionary history of this chromosome and characterized two human neocentromeres at 13q21, in search of information that could improve our understanding of the relationship between evolutionarily new centromeres, inactivated centromeres, and clinical neocentromeres.

**Results:**

Chromosome 13 evolution was studied, using FISH experiments, across several diverse superordinal phylogenetic clades spanning >100 million years of evolution. The analysis revealed exceptional conservation among primates (hominoids, Old World monkeys, and New World monkeys), Carnivora (cat), Perissodactyla (horse), and Cetartiodactyla (pig). In contrast, the centromeres in both Old World monkeys and pig have apparently repositioned independently to a central location (13q21). We compared these results to the positions of two human 13q21 neocentromeres using chromatin immunoprecipitation and genomic microarrays.

**Conclusion:**

We show that a gene-desert region at 13q21 of approximately 3.9 Mb in size possesses an inherent potential to form evolutionarily new centromeres over, at least, approximately 95 million years of mammalian evolution. The striking absence of genes may represent an important property, making the region tolerant to the extensive pericentromeric reshuffling during subsequent evolution. Comparison of the pericentromeric organization of chromosome 13 in four Old World monkey species revealed many differences in sequence organization. The region contains clusters of duplicons showing peculiar features.

## Abstract 2

The mammalian evolutionary history of chromosome 13 was characterized and evolutionary-new centromeres compared to two human neocentromeres at 13q21 using chromatin immunoprecipitation and genomic microarrays

## Background

Human neocentromeres are functional, analphoid centromeres that emerge epigenetically in ectopic chromosomal regions [1-4]. In the majority of cases, neocentromeres appear to rescue the mitotic stability of acentric chromosomal fragments, often giving rise to aneuploidy [5]. Studies on the evolutionary history of human chromosomes have shown that the centromere can reposition along the chromosome without marker order variation or leading to aneuploidy. This phenomenon is known as 'centromere repositioning' (CR) and has been reported in primates [6-11], in non-primate placental mammals [12,13], marsupials [14], birds [15], and plants [16].

Recently, two cases of 'repositioned' centromeres in otherwise normal individuals were fortuitously discovered [11,17]. These two cases can be considered as evolutionary centromere repositioning 'in progress' [17].

Two human neocentromeres were cytogenetically mapped to duplicons that flanked an ancestral centromere in band 15q24-26 that inactivated before hominoid divergence [10]. Additionally, a neocentromere at 3q26.1 was found located in the same chromosomal domain where an evolutionarily new centromere appeared in the Old World monkey (OWM) ancestor [11]. These studies, which used relatively low resolution cytogenetic techniques, suggested an intriguing relationship between human neocentromeres, ancestral centromeres and evolutionarily new centromeres (repositioned centromeres), which could represent the same phenomenon at different stages of fixation.

Sequences underlining some human neocentromeres have been identified using chromatin immunoprecipitation and genomic microarray (ChIP CHIP) analysis [18-20]. However, despite these recent advances, hypotheses to comprehensively explain the neocentromere phenomenon remain elusive. Phylogenomic studies could eventually provide information about the features, mechanisms and processes of evolutionarily new centromere seeding and development. We report here the evolutionary history of chromosome 13, which exhibits the most extensive clustering of neocentromeres of any human chromosome [3-5]. We found that this chromosome has been exceptionally conserved in evolution and we identified a locus, corresponding to human chromosome band 13q21, where CR events independently occurred in the OWM and pig lineages, whose ancestors diverged at least 95 million years ago (Mya). To further delineate the relationship between human neocentromeres and evolutionarily new centromeres, we used high resolution ChIP CHIP technology to determine the position of two human neocentromeres located in band 13q21. This analysis showed that the neocentromeres do not colocalize with each other or with the OWM and pig evolutionarily new centromeres at the sequence level, but instead map within a few Mb of them.

## Results

### Chromosome 13 evolution

**Figure 1:**
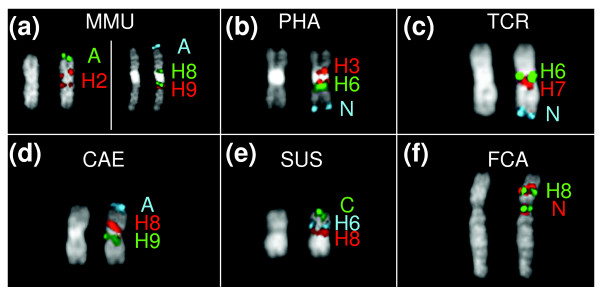
Examples of co-hybridization experiments on **(a) **macaque (MMU), **(b) **sacred baboon (PHA), **(c) **silvered-leaf monkey (TCR), **(d) **African green monkey (CAE), **(e) **pig (SUS), and **(f) **cat (FCA). The telomerically located probes in (a-e) were used for a correct identification of p and q arms of these metacentric chromosomes. The DAPI image alone is reported on the left to better show the morphology of the chromosome. Letters refer to BAC clones reported in Table 1.

**Figure 2:**
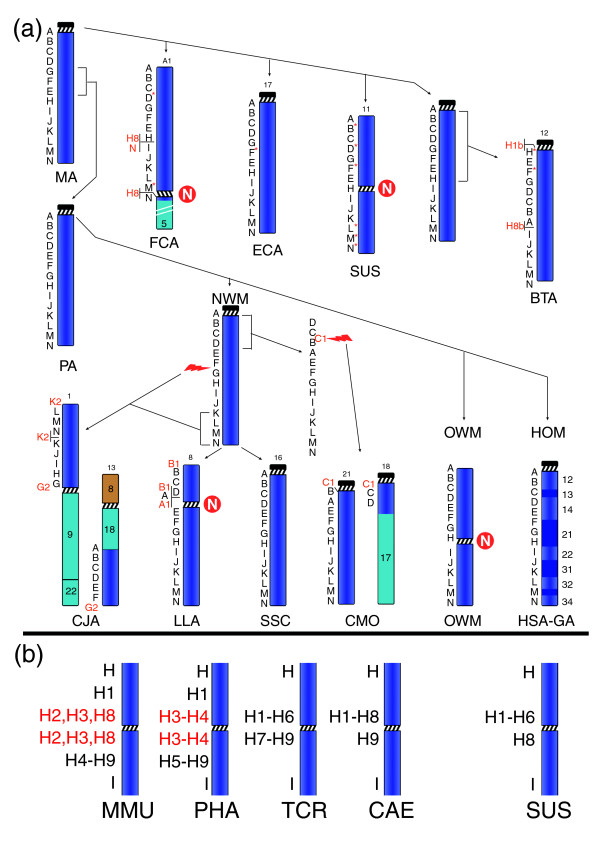
**Diagrammatic representation of the evolutionary history of chromosome 13**. **(a) **Marker order arrangement in the studied species, from which the arrangement of the mammalian ancestor (MA) and primate ancestor (PA) was derived (see text). N in a red circle stands for new centromere. The number that identifies the chromosome in each species is reported on top of the chromosome. The black letters on the left of each primate chromosome refer to the panel of BAC probes reported in Table 1 (human BACs); letters on cattle (BTA), pig (SUS), horse (ECA), and cat (FCA) chromosomes refer to BACs reported in Additional data file 1, obtained by library screening or from published databases (see text). Letters in red are the additional probes used to delimit chromosomal breakpoints or featuring unusual results (see N and H8 in the cat). Letter with asterisk indicate BACs identified on the radiation hybrids mapping data and used to fill gaps due to library screenings failure (see Table 1 and text). The long arm of cat chromosome A1 was shortened because of space constraint. The red lightning indicates chromosome break. **(b) **Results of FISH experiments of the H1 to H9 clones (Table 1) on OWM species (left) and in pig (SUS, right). Clones in red are duplicated. In OWM, the clones not reported in the figure failed to yield FISH signals. For details see text. (HOM = Hominoidea;HSA-GA = Homo Sapiens-Great Apes Ancestor).

Chromosome 13 evolution was studied by co-hybridizing a panel of 12 single-copy human BAC clones distributed along chromosome 13 (Table 1, clones in bold). The probes were hybridized on metaphase spreads of 11 primate species (see Materials and methods), including great apes and representatives of OWMs and New World monkeys (NWMs). Examples of fluorescence *in situ *hybridization (FISH) experiments are reported in Figure 1a-f. Mapping comparison, not considering the centromere, clearly showed that humans and ancestors of OWMs and NWMs share an identical marker order arrangement (Figure 2a).

This analysis was also performed in selected mammals for which BAC libraries were available. The sequence encompassed by each human BAC of the basic panel (in bold in Table 1) was searched for conservation against mouse and rat genomes. 'Overgo' probes were designed on the most conserved region of each BAC. The only exception was marker G, for which a highly conserved region was identified 2 Mb apart from the corresponding human marker. The overgo probes were then used to screen BAC libraries from cattle (CHORI-240 library), horse (CHORI-241), pig (CHORI-242), and cat (RPCI-86). This approach facilitated comparative mapping by assembling a panel of mammalian probes (Additional data file 1) orthologous to the loci encompassed by the human BACs. The identification of additional cattle probes took advantage of the collection of BAC clones positioned on the human sequence by BAC ends, as reported in Larkin *et al*. [21]. Results in non-primate mammals are also reported in Figure 2a, which shows the most parsimonious chromosomal changes necessary to reconstruct chromosome 13 evolution. In a comprehensive phylogenetic analysis of 64 species, Murphy *et al*. [22] defined four large superordinal clades of placental mammals, where Carnivora (cat (FCA)), Perissodactyla (horse (ECA)), and Cetartiodactyla (cattle (BTA), and pig (SUS)) belonged to clade IV and primates belonged to clade III. Remarkably, only a small inversion (markers E-F-G, Table 1, Figure 2a) distinguished the marker order of cat, horse, and pig (clade IV) with respect to primates (clade III).

### Centromere repositioning

The position of the centromere, operatively defined as the primary constriction of metaphase chromosomes, was found radically displaced, with respect to surrounding markers, in both OWM and pig (Figure 2a,b). We further refined the centromere position in OWMs using several human BACs spanning the interval H to I (BACs H1 to H9) that define a domain in band 13q21 of approximately 3.9 Mb (61,111,769 to 65,282,688 bp) (Table 1). FISH results (Figure 1a-f) showed that the centromeres in each of the four OWMs analyzed (*Macaca mulatta *(MMU), *Papio hamadryas *(PHA), *Trachypithecus cristatus *(TCR), and *Cercopithecus aethiops *(CAE)) were located in rather distinct chromosomal locations. In both MMU and PHA, several of these BACs were seen as duplicated signals on either side of the centromere. To better analyze the H1 to H9 region in pig (SUS), overgo probes were designed on the sequence of all H1 to H9 BACs. Only overgo probes from H1, H6, and H8 sequences provided positive results in library screening. Examples of co-hybridization FISH experiments using H6 and H8 probes on pig are reported in Figure 1e. These data showed that the pig centromere was also found in the region between probes H1 and H9 (Figure 2b). We can conclude that these CR events to the same chromosomal region were independent, because pig and OWMs diverged more than 90 Mya and belong to different mammalian clades. We extended our analysis to the elephant (*Loxodonta africana*, Afroteria, clade I), which is an outgroup with respect to clades III and IV [22]. We found that the elephant homolog to chromosome 13 was highly rearranged and provided no information on the original position of the centromere of the ancestral chromosome 13 or on the E-F-G inversion (data not shown).

Cat chromosome A1 is the result of a fusion of two chromosomes corresponding to human chromosomes 13 and 5 (Figure 2a). Marker order of the portion corresponding to human chromosome 13 was substantially conserved with respect to the mammalian ancestor (Figure 2a). In cat, markers N (distal telomeric in most species) and H8 (pericentromeric in OWM and pig) yielded duplicated signals at the FCA-A1 centromere and at the H8 locus (Figure 1f). In cattle (BTA), chromosome BTA12 contained a large paracentric inversion relative to the ancestral chromosome. The breakpoints of this inversion were mapped at the ancestral centromere (marker A) and between markers H and I, where the OWM and pig evolutionarily new centromeres are located.

### Chromosome 13 rearrangements in New World monkeys

Reiterative FISH experiments using additional human BAC clones were performed to more finely map the chromosome 13 fission and inversion breakpoints in NWMs (Table 1, Figure 2a). Human BAC probes that spanned breakpoints were identified by hybridization to both sides of the break on separate locations or chromosomes. In the dusky titi (CMO), the fission breakpoint was localized to probe C1 (BAC RP11-136G6, Table 1), which hybridized to the pericentromeric region of both CMO21 and CMO18 (Figure 2a). In the common marmoset (CJA, Callitrichinae), the fission breakpoint between markers F and G was localized to probe G2 (BAC RP11-939G7, Table 1), which hybridized to both the telomere of CJA5 and the centromere of CJA chromosome 1. The breakpoint of a subtelomeric inversion in CJA1 encompassing markers N-M-L was also mapped to probe K2 (RP11-351H1, Table 1), which hybridized to both the breakpoint and the telomere (Figure 2a).

In the wooly monkey (LLA), marker order was substantially conserved with respect to the NWM ancestor, except for the location of marker A between markers D and E, where an evolutionarily new centromere can be hypothesized (Figure 2a). Further analysis showed that a region of approximately 1.5 Mb delimited by markers B1 and A1 (Table 1) was found transposed from the pericentromeric region of the assumed primate ancestral chromosome to the new centromeric region of LLA8 (Figure 2a). Co-hybridization experiments suggested that the orientation of the segment toward the centromere is conserved with respect to humans. Marker B1 appeared to span the transposition breakpoint, while marker A1 was located adjacent to the heterochromatin/pericentromere boundary on the short-arm side. In this context it is worth noting that Cebidae (SSC and CJA) and Atelidae (LLA) diverged after the split of their common ancestor from Pitheciidae (CMO) [23].

### 13q21 neocentromeres

**Figure 3:**
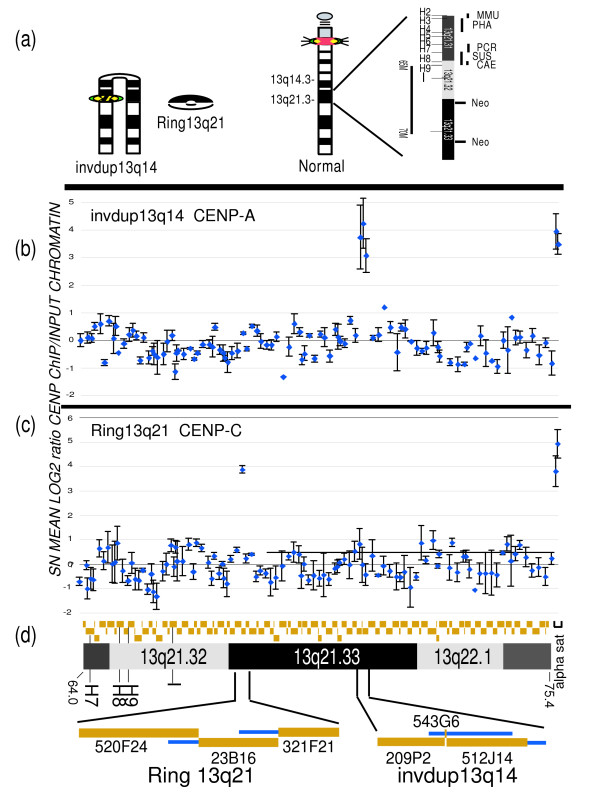
**ChIP CHIP results on the two neocentromere cases**. **(a) **Ideogrammatic representation of the neocentric chromosomes found in the two independent neocentromere cases. From left to right: the invdup13q14 chromosome with a neocentromere in band 13q21; the ring chromosome derived from band 13q21 to 13q22; a normal chromosome 13 for comparison. At the far right, the region 13q21.3 is expanded to show the relative position of BAC clones H2 to H9, along with the human neocentromeres and evolutionarily new centromeres of OWM and pig (see also Figure 2b). **(b) **ChIP on a CHIP analysis of the invdup13q14 chromosome using antibodies to CENP-A. **(3) **ChIP on a CHIP analysis of the ring13q21 chromosome using antibodies to CENP-C. For (b and c), the microarray was hybridized simultaneously with Cy-5 labeled CENP ChIP DNA (red) and Cy3 labeled input chromatin DNA (green). The scale normalized mean Log2 Cy-5:Cy-3 ratios and standard error for each BAC are shown plotted on the y-axis for three independent ChIP experiments. Alpha satellite DNA was included in the microarray as a positive control (far right). **(3) **Position of 107 contiguous BACs spanning 13q21 to 13q22 is shown across the x-axis. Positions of probes H7, H8, H9 and I (Table 1) are shown. Bottom: blow up of BACs that are positive for CENP proteins. Thin lines represent regions of BAC overlap. Scale in Mbp according to UCSC genome coordinates hg17 [Bioinformatics, #9821].

At least four independent cases of human neocentromeres have been observed in band 13q21, and thus we explored whether the position of these neocentromeres corresponds to the evolutionarily new OWM and pig centromeres. Three 13q21 neocentromeres have been observed on inverted duplication (invdup) chromosomes [5,24] and one on a small neocentric ring13q21 chromosome derived from a paracentric deletion of 13q21 (Figure 3a) [4,25]. The size of the ring13q21 neocentric chromosome (and thus the region that contains the neocentromere) was determined by FISH mapping to be approximately 11 Mb, bounded by the absence of BACs RP11-468L10 (chr13: 64.0 Mb) and BAC RP11-332E3 (chr13: 75.3 Mb) [25]. The neocentromere on an invdup13q14-qter chromosome (cell line 13a) [5] was confirmed to be located to this same approximately 11 Mb region by simultaneous FISH with BAC probes and immunofluorescence with antibodies to CENP-C (data not shown). This cytogenetic mapping showed that the 11 Mbp region containing the human 13q21 neocentromeres was overlapping with the locations of the CAE and SUS centromeres, between probes H7 and H9 (Table 1).

A genomic microarray (CHIP) was constructed containing 107 contiguous BACs (Figure 3d) spanning the entire ring13q21 chromosome, from BAC RP11-468L10 to RP11-332E3, inclusive. Neocentromere DNA from the invdup13q14 chromosome was obtained by chromatin immunoprecipitation (ChIP) from the cell line using antibodies to CENP-A. CENP-A ChIP DNA labeled with Cy-5 and input chromatin DNA labeled with Cy-3 were simultaneously hybridized to the genomic microarray, and positive BACs identified by the Cy-5/Cy-3 intensity ratios [19].

All BACs showed background ratios (log2 ≤ 1.19) except for three contiguous BACs (RP11-209P2 (log2 = 3.74 ± 1.16), -543G6 (4.23 ± 0.91), and -512J14 (3.07 ± 0.61)), which localized the CENP-A binding domain for this neocentromere (Figure 3b). The CENP-A ChIP protocol was technically not possible on the ring 13q21 cell line due to premature nuclear lysis and, therefore, an alternative ChIP protocol using antibodies to CENP-C was performed (see Materials and methods). All BACs showed background ratios (log2 ≤ 0.97) except for BAC RP11-23B16 (log2 = 3.87 ± 0.17), which localized the CENP-C binding domain for this neocentromere (Figure 3c). The primary data for the ChIP on CHIP analysis is provided in the Additional data files 7 to 17. Previous studies have shown that CENP-A and CENP-C co-immunoprecipitated onto the same chromatin at endogenous centromeres [26]. ChIP on CHIP analysis at a 13q32 neocentromere showed that the CENP-A and CENP-C chromatin domains colocalized at the resolution of a BAC array (Additional data files 2, 3, 16, and 17). Therefore, the use of either CENP-A or CENP-C will accurately identify the 13q21 neocentromere DNA using the BAC array.

Thus, these two human 13q21 neocentromeres occupied distinct genomic regions in 13q21.33 separated by approximately 3 Mb (Table 1). Furthermore, this analysis demonstrated that these 13q21 neocentromeres were separated from the OWM centromeric region by approximately 4 to 7 Mb (the position of the centromere in OWMs was assumed to be located in the middle of the H1 to H8 region, at approximately 63 Mb). The size of the CENP binding domains at these neocentromeres was estimated by removing overlapping regions of neighboring BACs that were negative; for example, the overlap of BAC RP11-520F4 and -321F21 was removed from BAC RP11-23B16 (Figure 3d). This may either over- or underestimate the CENP binding domain, depending on the size and resolution of the BAC clones and/or the sensitivity of the ChIP on CHIP. Like other neocentromeres [18-20], the CENP binding regions of these 13q21 neocentromeres, as estimated from the BAC array, were somewhat enriched in percent AT and LINE elements, and reduced in SINE elements relative to the genome averages (invdup13q, 182 kb, 65.0% AT, 30.61% LINE, 9.69% SINE; Ring, 52 kb, 66.1% AT, 22.41% LINE, 6.15% SINE).

### 13q21 gene content

Chromosome 13 has one of the lowest gene densities for any human chromosome [27]. We investigated the gene content of the approximately 3.9 Mb interval defined by the H1 to H9 clones (chr13: 61,178,154 to 65,079,597) harboring the OWM and pig evolutionarily new neocentromeres, by querying the specific tracks of the UCSC genome browser (UCSC, March 2006 release). RefSeq annotation does not report any gene in this region. AK127969 and AK098560, reported on the 'known genes' track appear as processed pseudogenes. Moreover, the region containing these RNAs is a portion of a duplicated sequence, with two palindromic copies in the region and a third copy at ch13:45,879,285-45,932,723. A copy of SATR2 satellite is localized in each of the three duplicated regions (chr13: 45,933,879 to 45,936,187, chr13: 63,306,505 to 63,309,036, chr13: 63,215,026 to 63,217,655). The genes PCDH20 (protocadherin 20, chr13: 60,881,821 to 60,887,282) and PCDH9 (protocadherin 9 isoform 1 precursor, chr13: 65,774,967 to 66,702,464) delimit this approximately 3.9 Mb gene desert.

Because of the association between ancestral centromeres and pericentromeric duplications [10,28], we examined the duplication content of the region corresponding to the evolutionarily new centromeres of 13q21 of humans in more detail (61 to 72 Mb). No enrichment of segmental duplications (>1 kb in length, >90% sequence identity) was observed within this particular region of 13q21 (0.5%) when compared to the chromosome 13 average (2.83%). To identify more ancient segmental duplications, we implemented an alternative approach based on whole genome assembly comparisons using BLASTZ (see Materials and methods), which facilitates the detection of shorter and less homologous duplications (>250 bp, >80% sequence identity). Based on this analysis, we identified an excess of short, more divergent pairwise alignments: the number of older duplications is five times that of more recent duplications, while the ratio when compared to the whole chromosome 13 for older duplications is two-fold (Additional data file 4). Sequence similarity searches of these divergent, short duplications show that more than 64% (51/79) of these regions correspond to exonic portions of other genes (ovostatin, olfactory receptors, and so on) or spliced ESTs, although the genes are not annotated as such within 13q21. We identified the remnants of intron-exon structure for 25 of these regions (19.2 kb) consistent with unprocessed pseudogenes, which were duplicated early during primate evolution (Additional data file 4). Several of the alignments show sequence homology to extant pericentromeric regions on human chromosomes 1p, 2p, 6p and 9q (Additional data files 5 and 6). When the interval is refined further to 127 kb (chr13: 63,188,927 to 63,316,389), the number of pairwise alignments increases several orders of magnitude when compared to the chromosome 13 average (18,354 alignments/Mb versus 161 alignments/Mb). However, the actual number of non-redundant duplicated base-pairs increases only moderately, suggesting that there is a limited amount of sequence that has been the target of several independent duplications. Although not definitive, these sequence properties are potentially consistent with an ancient pericentromeric region.

## Discussion

We have investigated the evolutionary history of chromosome 13 in 11 primate species and in selected non-primate mammals by analysis of marker order arrangement and centromere position, using FISH co-hybridization experiments of appropriate panels of BAC clones (Table 1, Figure 2a). If the centromere position is not taken into account, the marker order of the human chromosome 13 is perfectly conserved in squirrel monkey (SSC, NWM), in OWMs, and in hominoids. This form, therefore, is considered ancestral to primates. Cat (Carnivora), horse (Perissodactyla), and pig (Cetartiodactyla), belonging to mammalian clade IV, share substantially the same marker order arrangement as the primate ancestor except for the inversion of the region encompassed by markers E-F-G (Figure 2a). A limited number of additional inversions and translocations accounted for the chromosome 13 arrangements from the other analyzed mammalian species (Figure 2a).

Analysis of the elephant from mammalian clade 1 (Afrotheria), which branched from placental mammals about 105 Mya [29], was not informative in resolving the origin of chromosome 13. In this respect, however, it is worth noting that Svartman *et al*. [30] have reported that the human chromosome 13 painting library yielded a single signal on the chromosome 2 short arm of the Afrotherian short-eared elephant shrew (*Macroscelides proboscideus*), indicating that the chromosome was a unique entity in the Afrotheria ancestor.

The radiation hybrid data reported by Murphy *et al*. [12] did not detect the E-F-G inversion that we detected in cat, perhaps due to the limits of the radiation hybrid data set they used. The detailed physical map of the cow genome [31] is completely consistent with our data. The analysis of the chicken genome revealed that the entire euchromatic portion of human chromosome 13 is a continuous segment constituting approximately one-third of the chicken chromosome 1 long arm, in inverted orientation (chr1: 132,956,175 to 171,200,311) [32]; UCSC, February 2004 assembly). Within this segment there is an inversion whose breakpoint limits are, in the human sequence, within the intervals 40,284,579 to 40,380,292 bp (first breakpoint) and 51,628,385 to 51,849,306 bp (second breakpoint), which corresponds to the E-F-G inversion detected in non-primate mammals. Amazingly, these findings strongly suggest that the chromosome 13 marker order is shared by birds and mammals, although they diverged more than 250 Mya [32].

### Centromere repositioning

Hominoids and squirrel monkeys (SSC, NWM; clade III) and horse (clade IV) all have centromeres adjacent to marker A, which may represent a centromere position shared by the ancestor of primates (clade III) and Cetartiodactyla (clade IV). However, in all the studied OWMs (CAE, MMU, TCR, PHA; clade III) and in the pig (Cetartiodactyla, clade IV), the centromere was found to have repositioned to a region corresponding to the human 13q21 chromosomal region. The CR events were investigated in detail using additional probes spanning the region encompassed by markers H and I. The results show an extensive reshuffling of sequences around the centromere among the four studied OWM species (Figure 2b). The locations of the centromeres appear dispersed in an area of approximately 3.9 Mb with respect to the human sequence representing the ancestral form of the region. These centromeres are, very likely, the result of a single CR event in the OWM ancestor. Cercopithecinae/Colobinae diverged approximately 14.4 to 17.9 Mya [33], while the genera *Papio *and *Macaca *branched about 8.6 to 10.9 Mya [33]. The pericentromeric differences, therefore, provide evidence of an unprecedented degree of local centromeric/pericentromeric plasticity accompanied by extensive sequence remodeling. The position of the evolutionarily new centromere in pig falls within this region (Figure 2b). Mammalian clades III and IV diverged around 95 Mya [22], OWM and hominoids diverged approximately 25 Mya, and Cetartiodactyla (pig) and Perissodactyla (horse) diverged approximately 83 Mya [34]. We conclude that the pig centromere represents an independent centromere repositioning event in the same chromosomal region. This suggests that there are some features, conserved in the mammalian lineages for at least 70 million years, predisposing this region to centromere formation.

The observation that the region where the OWM and pig evolutionarily new centromeres were seeded is completely devoid of genes is a key finding. Studies of human neocentromere cases have shown that the neocentromere does not influence gene expression *per se *[35-37]. However, the subsequent heterochromatization of the region that invariably follows the evolutionarily new centromere seeding could, in theory, negatively affect gene expression. The finding that the relatively large H1 to H9 interval is completely devoid of genes may permit extensive sequence reshuffling, an inherent property of eukaryotic centromeres. We have recently reported that the centromere of OWM chromosome 3 is an evolutionarily new centromere that was generated after OWM divergence from Hominoidea [11]. Similarly to the present findings, the centromere seeding occurred within an approximately 430 kb region completely devoid of genes. It can be hypothesized, therefore, that the probability that an evolutionarily new centromere will become fixed during evolution is dependent upon the absence of nearby genes whose alteration due to the reshuffling could be selectively disadvantageous. Preliminary results of the characterization of the evolutionarily new centromere of macaque chromosome 6 are consistent with this model (our unpublished data).

The centromere of cat chromosome FCA-A1 was found located, with respect to the location of the ancestral mammalian centromere, at the opposite telomeric region, adjacent to marker N, where this chromosome fused with the homolog of human chromosome 5 (Figure 2a). This CR may represent a case of jumping centromeres, which is not a rare event in acrocentric chromosomes of primates [11]. Murphy *et al*. [12] have noted that telomere-to-centromere conversions are not rare in non-primate mammals. The cross-hybridization signals of H8 and N markers in cat, and the fact that the two breakpoints of the paracentric inversion in the cattle chromosome BTA12 fall in the centromeric region and in the region corresponding to the 13q21 in humans, suggest a special connection in mammals between the chromosome 13 ancestral centromere and the 13q21 region.

Additional potential CR events were observed on mammalian chromosomes in this study. On chromosome LLA8, the best explanation for the emergence of the centromere between markers D and E is the transposition of a 1.5 Mb region that contained the original centromere, which remained active (Figure 2a). This event would not represent true CR as previously defined [7]. However, it is possible that the transposition occurred after the CR event as a result of sequence movement from the inactivated, ancestral centromere to the newly formed centromere, as part of an accruing/degrading process. In this light, it is noteworthy that the transposed region is almost entirely composed of human pericentromeric segmental duplications.

### Human neocentromeres

To demonstrate a relationship between evolutionarily new centromeres and human neocentromeres, a chromosomal region must be found that contains both, as seen on human chromosome 3q26 [11]. Endogenous centromeres are relatively easy to localize in mammalian chromosomes using cytogenetic techniques due to the primary constriction and, more importantly, the large region of repetitive satellite DNA that inevitably forms there over evolutionary time [28]. However, human neocentromeres are more difficult to localize since they have not accumulated any repetitive DNA. Therefore, we used ChIP CHIP technology to precisely localize human neocentromeres and compared them to evolutionarily new centromeres.

Human chromosome 13 contains the highest number of reported neocentromeres of any human chromosome, which group into two major clusters at 13q21 and at 13q32 [4,5]. Thus, we examined the correspondence of human 13q21 neocentromeres with the OWM and pig chromosome 13 evolutionarily new centromeres. This analysis showed that two independent 13q21 neocentromeres were located approximately 4 Mb and 7 Mb distal to the OWM/pig centromeres. The present study, which used high resolution 'ChIP on a CHIP' technology (Figure 3) [19], did not demonstrate a precise co-localization between the neo- and evolutionarily new centromeres on 13q21. The relatively long distance between human neocentromeres and newly emerged evolutionary centromeres could be taken as evidence against a relationship between them. The two neocentromeres at 15q24-26 reported by Ventura *et al*. [10] were found to map about 10 Mb apart. This entire area, however, was shown to represent the wide chromosomal region where pericentromeric duplications of an inactivated centromere were dispersed following the chromosomal fission that generated chromosomes 14 and 15 [10]. As far as chromosome 13, the evolutionary history did not suggest any ancestral inactivated centromeres at 13q21. On the other hand, a significant enrichment of older (80% to 90%) segmental duplications corresponding to the exonic portions of genes was observed for a 127 kb portion of the region of 13q21. Although segmental duplications were seen throughout, the enrichment was most significant for this 127 kb segment.

This study adds two additional neocentromeres to the five that have been precisely mapped using chromatin immunoprecipitation [20]. However, comparative sequence analysis of these seven neocentromere sequences revealed no specific features in common to which neocentromerization competence could be ascribed. The centromere forming potential of specific genomic regions may reflect some relatively long-range property of the chromosomal domain, as opposed to the presence of specific sequence elements.

## Conclusion

The present study has tracked the extremely conserved evolution of human chromosome 13. The results defined important aspects of the complex scenario of centromere repositioning and human neocentromere emergence. The centromere of this chromosome repositioned in the same 13q21 region in OWMs and pigs, two species that diverged about 95 Mya. Fine-scale mapping of two clinical neocentromeres suggest that this propensity to form neocentromeres persists within the human population. The absence of genes in the region may be a critical component to progression/fixation of the novel centromere. Cross-species comparisons of chromosome 13 pericentromeric regions in OWM unveiled a striking reshuffling activity.

## Materials and methods

### Cell lines

Metaphase preparations were obtained from cell lines (lymphoblasts or fibroblasts) from the following species. Great apes: common chimpanzee (*Pan troglodytes*, PTR), gorilla (*Gorilla gorilla*, GGO), Borneo orangutan (*Pongo pygmaeus pygmaeus*, PPY-B). OWMs: rhesus monkey (*Macaca mulatta*, MMU, Cercopithecinae), sacred baboon (*Papio hamadryas*, PHA, Cercopithecinae), African green monkey (*Cercopithecus aethiops*, CAE, Cercopithecinae), silvered-leaf monkey (*Trachypithecus cristatus*, TCR, Colobinae). NWMs: wooly monkey (*Lagothrix lagothricha*, LLA, Atelinae), common marmoset (*Callithrix jacchus*, CJA, Callitrichinae), dusky titi (*Callicebus moloch*, CMO, Callicebinae), and squirrel monkey (*Saimiri sciureus*, SSC, Cebinae). Non-primate mammals: cattle (*Bos taurus*, BTA), horse (*Equus caballus*, ECA), pig (*Sus scrofa*, SUS), cat (*Felix catus*, FCA), and mouse (*Mus musculus*, MUS). EBV transformed human lymphoblasts containing the neocentric ring13q21 and invdup13q14 chromosomes were grown in standard RPMI media containing 10% fetal calf serum and antibiotics.

### FISH experiments

DNA extraction from BACs was reported previously [7]. FISH experiments were performed essentially as described by Lichter *et al*. [38]. Digital images were obtained using a Leica DMRXA2 epifluorescence microscope equipped with a cooled CCD camera (Princeton Instruments, Princenton, NJ, USA. Cy3-dCTP, FluorX-dCTP, DEAC, Cy5-dCTP and DAPI fluorescence signals, detected with specific filters, were recorded separately as gray scale images. Pseudocoloring and merging of images were performed using Adobe Photoshop™ software.

### Library screening

Sixteen overgo probes of 36 to 40 bp each were designed on sequences conserved between the human and mouse genomes according to the HomoloGene database [39] as described in [40]. The probes were hybridized to high-density filters of mammalian BAC libraries (see Results) and the images were analyzed with ArrayVision Ver 6.0 (Imaging Research Inc., Linton, UK) Linton, UK. The sequence and location of overgo probes, along with clones they identified, are reported in Additional data file 1.

The marker order reconstruction took advantage of the GRIMM software package [41], designed to outline the most parsimonious scenario of evolutionary marker order changes [42].

### Chromatin immunoprecipitation

CENP-A immunoprecipitation was performed as described in Alonso *et al*. [19]. CENP-C immunoprecipitation was performed using protocols modified from Oberley *et al*. [43]. Approximately 5 × 107 growing cells were crosslinked in 0.5% formaldehyde at room temperature for 10 minutes, followed by addition of glycine to 0.125 M for 5 minutes. Cells were washed in cold phosphate-buffered saline and incubated in 2 ml of lysis buffer (5 mM Pipes pH 8.0, 85 mM KCl, 0.5% w/v NP40, 1 mM PMSF = Phenylmethylsulfonyl fluoride and protease inhibitor cocktail (Sigma, Saint Louis, MO, USA) for 10 minutes at 4°C. Cells were centrifuged and resuspended in 1 ml of cold nuclei lysis buffer (50 mM Tris-HCl pH 8.1, 10 mM EDTA, 1% SDS, 0.5 mM PMSF and protease inhibitor cocktail (Sigma), and sonicated to obtain a DNA ladder ranging from 500 to 1,000 bp. The lysate was centrifuged for 10 minutes at 12,000 g, 4°C. The supernatant was adjusted to 1% Triton, 2 mM EDTA, 20 mM Tris-HCl, pH 8.1, 150 mM NaCl, 0.1% SDS, and precleared for 20 minutes at 4°C with 5 μg of rabbit IgG and 2% of blocked Protein G (Amersham Pharmacia Biotech, Piscataway, NJ, USA), followed by centrifugation. Then, 10 μl of rabbit polyclonal anti-CENP-C (Bill Earnshaw, Edinburgh, Scotland) were added and incubated for 4 h at 4°C. The immunocomplexes were recovered by incubation with 6% blocked ProtG for 2 h at 4°C and centrifugation. They were washed consecutively with low salt buffer (0.1% SDS, 1% Triton, 2 mM EDTA, 20 mM Tris pH 8.1, 150 mM NaCl) and high salt buffer (0.1% SDS, 1% Triton, 2 mM EDTA, 20 mM Tris pH 8.1, 500 mM NaCl), LiCl buffer (0.25 M LiCl,1% NP40, 1% deoxycholate, 1 mM EDTA, 10 mM Tris pH 8.1, and TE (10 mM Tris, 1 mM EDTA), and resuspended in 10 mM Tris HCl, pH 7.5, 5 mM EDTA, 0.25% SDS. To reverse the crosslinks, both input chromatin and immunocomplexes were adjusted to 200 mM NaCl and incubated at 60°C for 8 h, in the presence of 150 μg of Proteinase K PCR grade (Roche Applied Science, Indianapolis, IN, USA) and 20 μg of RNAseA (Qiagen, Valencia, CA, USA). DNA was phenol-chloroform extracted, ethanol precipitated with 1 μg of glycogen (Roche Applied Science) and quantified. Sonicated DNA (25 ng) from CENP-C input and immunocomplexes were repaired with 2.4 units of Kinase, 8 units of Klenow, and 8 units of T4 polymerase (NEB, Beverly, MA, USA) for 1 h at 37°C and ligation-mediated PCR using 2 ng per PCR reaction was carried out as described in Alonso *et al*. [19]. Ligation-mediated aminoallyl-dUTP PCR was carried out as described in [19].

### 13q21 BAC contig microarray construction and hybridization

The 11.4 Mb BAC contig containing BACs RP11-468L10 (AL356252) to RP11-332E3 (AL359392) was assembled by the UCSC Genome Bioinformatics group [44]. Microarrays were prepared as described in [19] by spotting sonicated BAC DNA onto aminosilane-coated glass slides (GAPSII, Corning, NY, USA). A plasmid containing chromosome 17-specific alpha satellite DNA was included as a positive control for ChIP. For microarray hybridization, 2.5 μg of amplified aminoallyl-dUTP PCR product was conjugated with approximately 20 ng of Mono-Reactive-Cy3 (input chromatin) or Mono-Reactive-Cy5 (ChIP) Dye Pack in 100 mM NaHCO_3 _(Amersham Biosciences, Little Chalfont, UK) for an hour in the dark. For hybridization of microarrays, 2.5 μg of each -Cy3 and -Cy5 labeled probe (specific activity approximately 60 nt/dye) were denatured 10 minutes at 72°C, with 14 μg of Cot, 575 μg of yeast tRNA and 50 μg of sonicated *Escherichia coli *DNA, in 25 μl of UltrahybTM (Ambion, Austin, TX, USA) followed by 2 h of annealing at 42°C. Slides were warmed to 42°C for half an hour, the hybridization mix placed on the slide, covered with a 22 × 22 LifterSlip (Erie Scientific Company, Porthsmouth, NH, USA) and incubated overnight at 42°C in an ArrayIt hybridization chamber (Telechem International Inc., Sunnyvale, CA, USA). The slides were washed in 50% formamide, 2 × SSC (SSC = solution of trisodium citrate and sodium clorure), 0.1% Tween-20 for 10 minutes at 45°C, followed by 2 × SSC, 0.1% Tween-20 for 15 minutes at 45°C, and 1× phosphate-buffered saine, 0.1% Tween-20 10 minutes at room temperature. For each microarray, the mean and standard deviation of the triplicate normalized ratios (Lowess) were calculated. Spots with a greater than 25% standard deviation (SD) from the mean were rejected (less than 3% of total spots). For each microarray a scale normalization was performed (X-mean of experiment/SD of experiment) as described by Smyth *et al*. [45]. Positive BACs were identified as those that were >3 SD from the mean and they represent 3 independent experiments.

To serve as negative controls, ChIP experiments were performed on a cell line containing a neocentromere in band 13q32 [19] and hybridized to the 13q21 BAC array. For anti-CENP-A, all BACs showed background ratios of log2 ≤ 0.88, indicating no CENP-A binding in the 13q21 region, while the alpha satellite DNA showed a ratio of log2 = 6.67. For anti-CENP-C, all BACs showed background ratios of log2 < 1.48, indicating no CENP-C binding in the 13q21 region, while the alpha satellite DNA showed a ratio of log2 = 5. All the primary data for the ChIP on CHIP analysis are provided in Additional data files 7 to 17.

### Segmental duplication analysis

We used a BLAST-based detection scheme [46] to identify all pairwise similarities representing duplicated regions (≥1 kb and ≥90% identity) within the finished sequence of chromosome 13 and compared to all other chromosomes in the NCBI genome assembly (May 2004, build 35). Divergence of duplication, and the number of substitutions per site between the two sequences, were calculated using Kimura's two-parameter method, which corrects for multiple events and transversion/transition mutational biases [47]. In order to detect more divergent duplications, a second all-by-all genome BLASTZ discontinuous search was performed within the finished genome to recover more divergent (>80%) and shorter (>250 bp) alignments (XS, unpublished data) [28].

## Additional data files

The following additional data are available with the online version of this paper. Additional data file  1 is a table listing non-primate mammalian BAC clones utilized in the study, and overgo probes used to screen them. Additional data file 2 is a figure showing ChIP on a CHIP analysis at a 13q32 neocentromere, indicating that the CENP-A and CENP-C chromatin domains colocalized at the resolution of a BAC array. Additional data file  3 contains the legend to the figure in Additional data file 2. Additional data file 4 is a table showing RefSeq genes with sequence similarity to 13q21duplicated regions. Additional data file 5 is a figure depicting segmental duplications (80% to 90% sequence identity and >1 kb or 250 bp) from a 11 Mb region (61 Mb to 72 Mb) of 13q21 to other regions of the genome. Additional data file 6 contains the legend to the figure in Additional data file 5. Additional data files 7 to 17 contain the primary data for the ChIP on CHIP analysis as follows. Additional data file 7 lists specifications of the custom made genomic BAC array (Array design). Additional data files 8, 9, 10, and 11 list primary data for three independent CenpA ChIP on a CHIP experiments and combined data for the three biological replicates for the cell line containing the invdup13q14 neocentric chromosome (cell line CHOP13). Additional data files 12, 13, 14, and 15 list primary data for three independent CenpC ChIP on a CHIP experiments and combined data for the three biological replicates for the cell line containing the ring13q21 neocentric chromosome (cell line Ring A). Additional data files 16 and 17 list data for control CENP-A and CENP-C ChIP performed on a cell line containing a 13q32 neocentromere (cell line BBB).

## Supplementary Material

Additional data file 1 Non-primate mammalian BAC clones utilized in the study, and overgo probes used to screen them Click here for file

Additional data file 2 The legend for this Figure is in Additional data file 3 Click here for file

Additional data file 3 Legend to the figure in Additional data file 2 Click here for file

Additional data file 4 RefSeq genes with sequence similarity to 13q21duplicated regions Click here for file

Additional data file 5 Segmental duplications (80% to 90% sequence identity and >1 kb or 250 bp) from a 11 Mb region (61 Mb to 72 Mb) of 13q21 to other regions of the genome Click here for file

Additional data file 6 Legend to the figure in Additional data file 5 Click here for file

Additional data file 7 Specifications of the custom made genomic BAC array (Array design) Click here for file

Additional data file 8 Primary data for three independent CenpA ChIP on a CHIP experiments and combined data for the three biological replicates for the cell line containing the invdup13q14 neocentric chromosome (cell line CHOP13) Click here for file

Additional data file 9 Primary data for three independent CenpA ChIP on a CHIP experiments and combined data for the three biological replicates for the cell line containing the invdup13q14 neocentric chromosome (cell line CHOP13) Click here for file

Additional data file 10 Primary data for three independent CenpA ChIP on a CHIP experiments and combined data for the three biological replicates for the cell line containing the invdup13q14 neocentric chromosome (cell line CHOP13) Click here for file

Additional data file 11 Primary data for three independent CenpA ChIP on a CHIP experiments and combined data for the three biological replicates for the cell line containing the invdup13q14 neocentric chromosome (cell line CHOP13) Click here for file

Additional data file 12 Primary data for three independent CenpC ChIP on a CHIP experiments and combined data for the three biological replicates for the cell line containing the ring13q21 neocentric chromosome (cell line Ring A) Click here for file

Additional data file 13 Primary data for three independent CenpC ChIP on a CHIP experiments and combined data for the three biological replicates for the cell line containing the ring13q21 neocentric chromosome (cell line Ring A) Click here for file

Additional data file 14 Primary data for three independent CenpC ChIP on a CHIP experiments and combined data for the three biological replicates for the cell line containing the ring13q21 neocentric chromosome (cell line Ring A) Click here for file

Additional data file 15 Text: Primary data for three independent CenpC ChIP on a CHIP experiments and combined data for the three biological replicates for the cell line containing the ring13q21 neocentric chromosome (cell line Ring A) Click here for file

Additional data file 16 Data for control CENP-A and CENP-C ChIP performed on a cell line containing a 13q32 neocentromere (cell line BBB) Click here for file

Additional data file 17 Data for control CENP-A and CENP-C ChIP performed on a cell line containing a 13q32 neocentromere (cell line BBB) Click here for file

## Figures and Tables

**Table 1:** Human probes used in the study

Code	BAC	Accession no.	Map	UCSC (May 2004)	Duplicons >20 kb (kb)
*A1*	RP11-631L24	BES	13q11	18,200,927-18,369,699	
**A**	**RP11-110K18**	AL137119	13q12.11	19,404,213-19,546,706	
*B1*	RP11-264J4	AL138688	13q12.11	19,546,607-19,674,400	20,660,153-20,838,143 (178 kb)
**B**	**RP11-45B20**	AL445985	13q12.12	23,305,109-23,483,639	23,779,363-24,076,626 (279 kb)
					24,400,396-24,504,215 (104 kb)
*C1*	RP11-136G6	BES	13q12.2	27,400,228-27,581,446	
**C**	**RP11-64I8**	AL158065	13q12.3	30,502,889-30,571,189	
**D**	**RP11-142E9**	BES	13q13.2	33,252,754-33,451,136	34,390,618-34,605,903 (215 kb)
**E**	**RP11-145J3**	AL137878	13q14.11	42,340,023-42,427,380	40,186,105-40,396,852 (210 kb)
					40,892,985-40,914,548 (21 kb)
					41,919,710-41,988,111 (68 kb)
**F**	**RP11-30N18**	BES	13q14.12	44,481,780-44,541,540	
*F1*	RP11-661C17	BES	13q14.13	45,660,985-45,826,015	
*G2*	RP11-939G7	BES	13q14.13	45,754,269-45,939,953	45,879,285-45,940,396 (61 kb)
*G1*	RP11-945G11	BES	13q14.13	45,928,366-46,127,167	
**G**	**RP11-103J18**	AL138875	13q14.2	48,711,312-48,801,118	51,634,782-52,115,918 (481 kb)
**H**	**RP11-10O23**	AC013618	13q21.1	55,430,662-55,603,003	56,611,299-56,645,585 (34 kb)
*H1*	RP11-543A19	AL590102	13q21.2	61,111,769-61,178,154	
*H2*	RP11-1043D14	BES	13q21.31	61,282,357-61,458,258	
*H3*	RP11-539I23	AL354803	13q21.31	61,530,165-61,709,544	
*H4*	RP11-527N12	AL354810	13q21.31	62,520,878-62,699,203	
*H5*	RP11-320N6	AL359208	13q21.31	62,804,903-62,944,551	
*H6*	RP11-520F9	AL355879	13q21.32	63,407,676-63,481,486	63,188,927-63,316,389 (127 kb)
*H7*	RP11-318C5	AL356253	13q21.32	64,037,783-64,238,420	
*H8*	RP11-379K8	AL354739	13q21.32	64,786,660-64,966,449	
*H9*	RP11-612P16	BES	13q21.32	65,079,597-65,282,688	
**I**	**RP11-187E23**	AL136999	13q21.32	66,092,979-66,264,337	
NC ring	RP11-23B16	AL161894	13q21.33	67,841,134-67,947,116	
NC invdup	RP11-209P2	AL162212	13q21.33	70,669,808-70,794,225	
	RP11-543G6	AL590141	13q21.33	70,794,126-70,797,735	
	RP11-512J14	AL354995	13q21.33	70,797,636-70,947,217	
**K**	**RP11-188A23**	AL354831	13q22.3	77,153,157-77,297,742	
*K2*	RP11-351H1	BES	13q31.1	84,396,772-84,582,561	
**L**	RP11-143O10	AL353635	13q31.2	88,642,642-88,673,975	
**N**	**RP11-245B11**	AL161774	13q34	113,770,458-113,932,864	91,231,475-92,135,792 (894 kb)
tel				114,142,980	111,979,464-112,007,994 (28 kb)

Probes in bold were used to characterize all primate species. Probes in italics were used to define specific rearrangements. BES, BAC ends; NC, neocentromere.
