# Supplementary material for: Independent centromere formation in a capricious, gene-free domain of chromosome 13q21 in Old World monkeys and pigs
Source: Genome Biol. 2006 Oct 13;7(10):R91. doi: 10.1186/gb-2006-7-10-r91 (PMC1794570; doi:10.1186/gb-2006-7-10-r91)
Supplement: Additional data file 1 — Non-primate mammalian BAC clones utilized in the study, and overgo probes used to screen them [file gb-2006-7-10-r91-S1.doc]

**Supplemental Table 1** Non-primate mammalian BAC clones utilized in the study, and overgo probes used to screen them

| **Probe** | **Cat**  RPCI-86 | **Horse**  CHORI-241 | **Cattle**  CHORI-240 | **Pig**  CHORI-242 | **Overgo sequence** | **UCSC May 2004** |
| --- | --- | --- | --- | --- | --- | --- |
| **A** | 274G5 | 474K9 | 49E16 | 79K11 | TGACAACTTGTACTGGTTGCCGAACACAGTGCAGGTTTTT | 19,498,765-19,498,804 |
| **B** | 358I19 | 274D19 | 187E2 | - | ACACTGAAGTGGATTTTCAAGGACTTGACTTGCCCAAGCT | 23,044,760-23,044,799 |
| **C** | 218H14 | 244I22 | 150D1 | 109B14 | GGAGACTGTGAGGTTTGTTGAGCATCAGTTTGTACCTGGG | 30,615,981-30,616,020 |
| **D** | - | 251G23 | 72C5 | - | CCTAGGATTAACTTCAAGGTGGTAGTTACTTGCAATGGTG | 33,296,083-33,296,122 |
| **E'** | - | - | 71J20† | 150N22 | CAGCAGTAGCTGGAGCAGTCTTGGTTTAGAAGGAGA | 41,785,192 - 41,785,227 |
| **E** | 208B14 | 314H18 | 44A14† | - | ATGCTTTTCTGGACCGACTCCAAGGCAAAAGTCAACCAGG | 42,367,197-42,367,236 |
| **F** | 460C22 | - | - | - | GCATGAGTGTAACCATGGAGACCCATTGAAACCAGC | 44,520,244-44,520,279 |
| **G** | 195B13 | 54H9 | 114F23 | 32P17 | AGGGAAGACTTGAGTCAGGGTCAGGAGGAGGAAACTGGAT | 46,075,423-46,075,462 |
| **G'** | - | - | - | 45D15 | TCCTGCTCAGGTTCCAATGATGTCCCCAAATGGTTC | 48,586,793 - 48,586,828 |
| **H** | 201J20 | 295B13 | - | 49B1 | GTTGTCTGTGGAAGATGTAACCACAATGGTGCTGTG | 56,326,117-56,326,152 |
| **H1** | 231F6 | 253K6 |  | 50J4 | GAACTCAACCTATACCAAAGGAACAGCTTTCTCACC | 59,384,240-59,384,275 |
| **H1b** |  |  | 110D16† |  |  | 60,961,661-60,961,873 |
| **H6** | 207D17 | 242J8 | - | 75J14 | CTCAGCATGTTCAGCATGCTTAATGAACTCCTTGGG | 63,152,879-63,152,914 |
| **H8** | 201I1 | 359D17 | 67I22 | 112O17 | CATGAGTGGTTTATGGAAGTTGAGATATGAGCTGAGTATG | 64,874,777- 64,874,816 |
| **H8b** |  |  | 25A4† |  |  | 65,275,235-65,276,254 |
| **I** | 298A16 | 360K5 | 121L6† | RP11-187E23* | ATGCCTGAGTGCTATAGAGCTGAAGTAATTGCACTC | 66,128,079-66,128,114 |
| **K** | 201C12 | 303L7 | 53K13 | CHORI240-53K13* | TCTCCATCAAGGGCAACTCTCTCAGAGGGCAAACAAAAGC | 77,670,695-77,670,734 |
| **L** | 250E20 | 463N7 | 289M4 | - | TAGGTCTATGGGACCTTGGCCAAAGTCAGCATAATGAGGT | 88,246,713-88,246,752 |
| **M** | - | 250G18 | 60M16 | - | TGAAGTGCCAGTTCAGCTGTGAAGTGGTGTGATGTGATGC | 103,590,913-103,590,948 |
| **N** | 289I10 | 300J2 | 130L11 | - | GGGAACCTTTGTTGAACAACTTGGGGCATGTCTGCAGAAA | 114,045,480-114,045,519 |

* Probes from other species successfully used in pig.

† Probes whose mapping is reported by Larkin et al. (2003) database (see text). The original position on the UCSC June 2002 release has been converted to the May 2004 release.
