# Supplementary material for: Independent centromere formation in a capricious, gene-free domain of chromosome 13q21 in Old World monkeys and pigs
Source: Genome Biol. 2006 Oct 13;7(10):R91. doi: 10.1186/gb-2006-7-10-r91 (PMC1794570; doi:10.1186/gb-2006-7-10-r91)
Supplement: Additional data file 3 — Legend to the figure in Additional data file 2 [file gb-2006-7-10-r91-S3.doc]

Supplemental Figure 1 Legend

**Colocalization of CENP-A and CENP-C at a 13q32 neocentromere.**

A genomic microarray containing 126 contiguous BACs spanning 14Mbp on band 13q32 was constructed as described (19). This microarray was used to analyze cell line BBB, which contains a supernumerary invdup 13q21 chromosome with a neocentromere in band 13q32 (19). Results of ChIP on a CHIP showed that both CENP-A and CENP-C colocalized to the same BAC RP11-46I10. A) ChIP on a CHIP analysis of cell line BBB using antibodies to CENP-A. n=3 BAC RP11-46I10: mean log2 ratio SN = 6.45±0.31se, BAC RP11-29B2: mean log2 ratio SN = 2.12±0.37se, alpha satellite: mean log2 ratio SN =7.32±0.53se, all other BACs mean log2 ratio SN≤1.13±0.52se B) ChIP on a CHIP analysis of cell line BBB using antibodies to CENP-C. n=3, BAC RP11-46I10: mean log2 ratio SN = 4.66±0.92se, BAC RP11-29B2: mean log2 ratio SN = 1.18±1.2se, alpha satellite: mean log2 ratio SN = 6.42±0.39se; all other BACs mean log2 ratio SN≤0.96±0.52se.The red dashed line in each graph represents the experimental mean log2 ratio SN plus three times the standard deviation (sd). BACs above the line were considered positives.
