# Supplementary material for: Independent centromere formation in a capricious, gene-free domain of chromosome 13q21 in Old World monkeys and pigs
Source: Genome Biol. 2006 Oct 13;7(10):R91. doi: 10.1186/gb-2006-7-10-r91 (PMC1794570; doi:10.1186/gb-2006-7-10-r91)
Supplement: Additional data file 6 — Legend to the figure in Additional data file 5 [file gb-2006-7-10-r91-S6.doc]

Supplemental Figure 2 Legend

The image depicts segmental duplications (80-90% sequence identity and > 1 kb or 250bp) from a 11 Mb region (61 Mb-72 Mb) of 13q21 to other regions of the genome. Several of these more ancient interchromosmal duplications map to pericentromeric regions on chromosomes 2p, 6p, 9q, etc.
